# Artificial Intelligence in Routine IVF Practice

**DOI:** 10.3390/biology15010042

**Published:** 2025-12-26

**Authors:** Grzegorz Mrugacz, Aleksandra Mospinek, Małgorzata Jagielska, Dariusz Miszczak, Anna Matosek, Magdalena Ducher-Hanaka, Paweł Gustaw, Klaudia Januszewska, Aleksandra Grzegorczyk, Svetlana Pekar

**Affiliations:** 1 Center for Reproductive Medicine Bocian, Białystok Branch, 26 Akademicka St., 15-267 Białystok, Poland; 2 Center for Reproductive Medicine Bocian, Łódź Branch, 157a Piotrkowska St., 90-440 Łódź, Poland; 3 Center for Reproductive Medicine Bocian, Warszawa Branch, 2A Stawki St., 00-193 Warszawa, Poland

**Keywords:** artificial intelligence, IVF, embryo selection, clinical validation, ethics, interoperability

## Abstract

**Background**: Artificial Intelligence (AI) has emerged as a transformative tool in in vitro fertilization (IVF) as it has done in other sectors. In IVF, AI offers advancements in embryo selection, treatment personalization, and outcome prediction. It does so by leveraging deep learning and computer vision, as well as AI-driven platforms such as ERICA, iDAScore, and IVY where the goal is to address the limitations of traditional embryo assessment. Key amongst them are the issues of subjectivity, labor intensity, and limited predictive power. Despite rapid technological progress, the integration of AI into routine IVF practice faces key challenges. These are issues related to clinical validation, ethical dilemmas, and workflow adaptation. **Rationale/Objectives**: This review synthesizes current evidence to evaluate the role of AI in IVF, focusing on six critical dimensions: (1) the evolution of AI from traditional embryology to algorithmic assessment, (2) clinical validation and regulatory considerations, (3) limitations and ethical challenges, (4) pathways for clinical integration, (5) real-world applications and outcomes, and (6) future directions and policy recommendations. The objective is to provide a comprehensive roadmap for the responsible adoption of AI in reproductive medicine. **Outcomes**: AI demonstrates significant potential to improve the precision and efficiency of IVF. Studies report that AI models can achieve 10 to 25% higher accuracy in predicting embryo viability and implantation potential compared to traditional morphological assessment by embryologists. This enhanced predictive power supports more consistent embryo ranking, facilitates elective single-embryo transfer (eSET) strategies, and is associated with 30 to 50% reductions in embryologist workload per embryo cohort. Early adopters report promising trends. However, large-scale randomized controlled trials have yet to conclusively demonstrate a statistically significant increase in live birth rates per transfer compared to expert embryologist selection. The most immediate and evidenced value of AI lies in hybrid decision-making models. This is where it augments embryologists by providing data-driven, objective support, thereby standardizing workflows and reducing subjectivity. **Wider Implications**: The sustainable integration of AI into IVF banks on three key aspects: robust evidence generation, interdisciplinary collaboration, and global standardization. To foster these, policymakers ought to establish regulatory frameworks for transparency and bias mitigation. On their part, clinicians need training to interpret AI outputs critically. Ethically, safeguarding patient trust and equity is non-negotiable. Future innovations, mainly AI-enhanced genomics and real-time monitoring, could further personalize care. However, their success depends on addressing current limitations. By balancing innovation with ethical vigilance, AI holds the potential to revolutionize IVF while upholding the highest standards of patient care.

## Simple Summary

Choosing the best embryo for transfer during in vitro fertilization (IVF) is a difficult and subjective task. Embryologists currently examine embryos under a microscope. However, this method can be inconsistent and does not always identify the embryo most likely to result in a pregnancy. This review examines how Artificial Intelligence (AI) is being used to help solve the dilemma. AI can analyze thousands of images and videos of developing embryos to spot subtle signs of health that humans might miss. It acts as a powerful assistant to embryologists. Mainly, it helps them make more consistent, data-driven choices. Indeed, AI shows great promise in making IVF more efficient and reducing the workload for clinic staff. Nevertheless, we find that challenges remain. Key amongst them is the need for fair and unbiased algorithms and clear rules for how these tools ought to be used in clinics. The responsible integration of AI has the potential to make fertility treatments more successful, personalized, and accessible to patients worldwide.

## 1. Introduction

Artificial Intelligence (AI) has emerged as a transformative force in healthcare [1]. Its application in reproductive medicine has been just as significant as it has been in other domains. In in vitro fertilization (IVF), AI-driven platforms promise significant advancements in embryo selection precision, treatment personalization, and outcome prediction [2,3,4,5]. All these have the potential to revolutionize success rates, reduce subjectivity, and enhance patient experiences.

The current landscape features sophisticated deep learning and computer vision algorithms. Key examples include ERICA and iDAScore platforms, amongst others [6,7,8]. Their focus is on analyzing embryo morphology, developmental kinetics, and viability biomarkers. Nevertheless, despite rapid commercial adoption, real-world evidence of efficacy remains scarce. Key factors and influencers such as variability in validation protocols, dataset diversity, and clinical integration challenges highlight a critical gap between AI’s true technological potential and routine implementation [9,10,11,12,13,14]. Hence, this paper posits that the sustainable integration of AI into IVF demands more than algorithmic sophistication. It necessitates robust clinical validation frameworks, proactive ethical governance, and structured implementation strategies to ensure safety, equity, and efficacy.

We examine this paradigm through six critical dimensions. The first is the evolution of AI in IVF, tracing its emergence from traditional embryology to automated systems. The second topic is AI’s clinical validation, scrutinizing regulatory standards and evidence gaps. Thirdly, this paper will cover pertinent AI limitations and ethics, addressing biases, data privacy, and moral dilemmas in automated selection. This paper will then preview significant pathways to clinical integration, outlining training, interoperability, and workflow adaptation, and reviewing real-world case studies, highlighting successes and barriers across global clinics. We will then conclude by reviewing policy and future directions, advocating for standardized frameworks and AI-augmented innovations.

By accomplishing all the above through the synthesis of recent evidence and emerging debates, this review provides a roadmap for ethically grounded, evidence-based AI adoption in reproductive medicine.

## 2. The Evolution of AI in IVF: From Subjective Morphology to Algorithmic Assessment

### 2.1. Traditional Methods of Embryo Assessment and Limitations

Traditionally, embryo selection in IVF relied and continues to bank on morphological assessment. This is usually by trained professionals (embryologists), who leverage static microscopy. With the traditional approach, embryos are evaluated at specific timepoints such as day 3 for cleavage stage or day 5/6 for blastocysts. These evaluations are based on criteria like cell number, symmetry, fragmentation, blastomere size, blastocyst expansion, trophectoderm (TE) quality, and inner cell mass (ICM) quality [15,16,17]. To improve assessment efficacy, standardized grading systems such as Gardner and the Istanbul consensus were developed. However, the traditional assessment remained and continues to be inherently subjective and qualitative. Studies have repeatedly demonstrated key limitations such as the significant inter- and intra-observer variability. This is where embryologists frequently disagreed on the grading and implantation potential of the same embryo [15,16,18,19,20]. This subjectivity, as insinuated, underlay a major limitation in consistently identifying embryos with the highest implantation potential. This is crucial for elective Single-Embryo Transfer (eSET) strategies aimed at reducing multiple pregnancies [16,19,21].

A second key limitation of the traditional assessment approach is its static nature. The brief observations alongside morphology scores missed dynamic developmental cues such as cleavage anomalies and multinucleation that are only visible through continuous monitoring [5,22]. Time-lapse microscopy (TLM) was developed to address this key limitations. It functions by capturing continuous development and revealing morphokinetic parameters such as the timing of cleavages and blastocyst formation [4,5,16,23]. However, even with the advent of TLM, traditional embryo assessment still faced operational challenges, some due to TLM’s adoption. For instance, with more data due to TLM, embryologists’ interpretation of morphokinetics remained complex, adding another layer of subjectivity [4,5,23].

Another key limitation of traditional morphology is limited predictive power for complex outcomes like live birth [16,19,22]. Pertinent models that attempted to predict outcomes based on morphology and patient factors often exhibited insufficient accuracy for relevant clinical decision-making [15,23,24]. Traditional assessment was also hindered by its labor-intensive nature. The manual assessment and scoring fundamentally limited scalability in high-volume clinics [5,23]. Lastly, traditional methods lacked sufficient tools for non-invasive ploidy assessment. The method relied heavily on invasive Preimplantation Genetic Testing for Aneuploidy (PGT-A). As is extensively highlighted in the literature, PGT-A has its own limitations, costs, and ethical debates [17,25].

### 2.2. Introduction of AI into Reproductive Technologies

The key limitations of the traditional assessment approach have fostered the introduction of Artificial Intelligence (AI), particularly machine learning (ML) and deep learning (DL), into reproductive technologies [1]. The core promise was to leverage computational power to objectively analyze complex datasets beyond evidenced human capacity. As such, the outcome would be an improved prediction accuracy and consistency [2,3,4,5,17,26,27].

The core focus of the initial integration was to automate and enhance the analysis of images and videos. Since their introduction to improve on the manual assessment processes, TLM systems generated vast amounts of image sequence data. The quantity of data was ideal for computer vision techniques. In early AI interventions, the focus was to automate basic tasks like embryo stage detection and cell counting. This would have in turn reduced the potential errors and the workload of embryologists [4,5,17]. However, researchers quickly realized AI’s true potential, which is to identify subtle patterns and features invisible to the human eye within TLM-generated images. These include nuances in texture, shape changes over time, or specific morphokinetic signatures correlating with embryo viability or chromosomal status [4,5,15,17,23,24]. Hence, immediately with its introduction, the objective shifted from simply replicating embryologists to discovering novel predictive biomarkers [15,17].

AI, even in its early phase in the field, leveraged multimodal data integration. That is, beyond the images, pertinent models began incorporating diverse data types. These included patient demographics such as age and BMI, ovarian stimulation parameters such as hormone levels, sperm parameters, endometrium receptivity markers that include ultrasound features and transcriptomics, and even proteomic and metabolomic profiles of the embryo culture medium [2,27,28,29]. The objective with this integration was to build comprehensive predictive models that advanced beyond the isolated embryo assessment [2,27,29]. AI also offered potential for non-invasive ploidy assessment (NIPA). By analyzing TLM images or other non-invasive markers, AI algorithms aimed to predict embryo chromosomal status. This had been theorized to potentially reduce the need for invasive PGT-A biopsies in some cases [15,17,25]. Furthermore, AI tools were developed to personalize treatment protocols [27,28] or even suggest the optimal day for transfer or the best sperm for injection (ICSI) [3,17]. The overarching aim was to improve decision-making at multiple points in the IVF pathway. This would ultimately increase efficiency, standardization, and success rates while reducing costs and risks like OHSS or multiple pregnancies [3,17,26,30].

### 2.3. Overview of Key AI Platforms and Commercial Tools Currently in Use

There are several commercially available platforms integrated into clinical workflows, better highlighted in Table 1 below. These tools primarily focus on embryo assessment and selection by leveraging TLM data. They include ERICA (Embryo Ranking Intelligent Classification Algorithm), a deep learning algorithm trained on large datasets of TLM videos and clinical outcomes. ERICA leverages morphokinetics and visual features to rank embryos based on key markers. These are predicted blastocyst formation, implantation potential, and live birth probability [24]. There is also iDAScore (Vitrolife, Gothenburg, Sweden), a commercial AI scoring system integrated within Vitrolife’s TLM systems (EmbryoScope+) [17]. This system combines traditional morphokinetic parameters and deep learning-derived image features. With this, it capably generates a continuous score predicting blastocyst formation and implantation potential. The third key platform is LifeWhisperer Viability (Presagen, San Francisco, CA, USA). It leverages both static images and TLM data. Its pertinent AI model assesses embryo images to predict clinical pregnancy likelihood [24]. It is also designed to be compatible with images taken on standard microscopes, increasing accessibility [24].

Another key platform is IVY (Fairtility, Tel Aviv-Yafo, Israel). Labeled as glass AI, its transparency provides explainable assessments. IVY analyzes TLM data to predict key developmental milestones such as blastulation and implantation potential. At the same time, it also offers quality control metrics for the lab environment as noted in Glatstein et al. There are also AI Models for Non-Invasive Ploidy Assessment (NIPA). Several AI tools such as STORK-A are being developed. These tools analyze TLM images or blastocyst morphology to predict chromosomal status. Through such, they offer a potential alternative or triage tool before PGT-A [15]. In their study, Barnes et al. [15] highlighted a non-invasive AI model predicting blastocyst ploidy with high accuracy using static images and clinical data. Lastly, there are also ovarian stimulation and protocol personalization tools. These platforms can effective predict ovarian response based on patient characteristics, biomarkers such as AMH AFC, and stimulation parameters. In doing so, they aid in dose personalization and cycle planning [27,28,29,31,32,33,34,35]. Table 1 below clearly highlights the variance in these platforms.

**Table 1:** A summary of commercially available AI platforms currently integrated into clinical workflows.

Platform	Category	Developer	Key Technology	Functionality	Key References
ERICA	Embryo Viability Assessment	Chavez-Badiola et al. (2020)	Deep Learning (CNN/RNN)	Analyzes TLM videos to rank embryos by predicted: Blastocyst formation Implantation potential Live birth probability Integrated with specific TLM incubators	[24,33,36]
iDAScore	Embryo Viability Assessment	Vitrolife	Hybrid model: Traditional morphokinetics DL image features	Generates continuous score predicting: Blastocyst formation Implantation potential Fully integrated with EmbryoScope+ TLM systems	[6,7,17,36]
LifeWhisperer Viability	Embryo Viability Assessment	Presagen	Deep Learning (image analysis)	Assesses static images (day 5 blastocysts) to predict: Clinical pregnancy likelihood Compatible with standard microscopes	[33,36]
IVY	Embryo Viability Assessment	Fairtility	Transparent AI (“Glass AI”) Explainable DL	Analyzes TLM data to predict: Developmental milestones (blastulation) Implantation potential Lab QC metrics	[36]
STORK-A	Non-Invasive Ploidy Assessment (NIPA)	Research groups	Deep Learning plus Clinical data fusion	Predicts chromosomal status (ploidy) non-invasively via: TLM image analysis Blastocyst morphology PGT-A triage tool	[15,33,36]
Ovarian Stimulation AI such as Alife Health	Ovarian Stimulation & Protocol Personalization	Alife Health et al.	Machine Learning regression models	Predicts ovarian response: Oocyte yield Personalizes: Stimulation protocols Medication dosing Uses: Patient demographics Biomarkers (AMH, AFC)	[27,28]

### 2.4. Key Trends and Considerations

One key aspect is integration. Top tools are increasingly being integrated directly into TLM incubators or Laboratory Information Management Systems (LIMS). Through this, they effectively streamline workflow [5,8,34]. Regarding validation, there is a critical push for pertinent studies, more so on diverse patient populations, to prove generalizability beyond the training data [6,9,24,34]. Further, there is a growing demand for tools that explain why an embryo received a certain score. This is necessary to foster the transition beyond opaque predictions to build trust and facilitate human–AI collaboration [31,32,33,37,38]. Clinically, studies are beginning to show that AI-assisted selection can improve consistency. Further, their operational efficiency outperforms embryologists in predicting implantation and live birth [8,23,24,31]. However, long-term impact on cumulative live birth rates and cost-effectiveness is yet to be fully established [24,34,39].

The evolution of AI in IVF underlies a paradigm shift towards data-driven, algorithmic decision support. Even in the present, traditional morphology and embryologist expertise remain fundamental. However, AI tools are rapidly maturing into powerful assistants. These tools enhance objectivity, efficiency, and potentially improve outcomes across the IVF pathway [3,5,12,17,27,30]. Their successful integration depends on rigorous validation and ethical consideration. It is also dependent on seamless collaboration between technology and clinical expertise [33,38,40].

## 3. Clinical Validation of AI in IVF: Bridging the Promise and Practice

The integration of AI into IVF has immense potential. However, its translation into routine clinical practice significantly depends on rigorous clinical validation. This section of the review evaluates the ability of AI tools to perform reliably, safely, and effectively in real-world settings.

### 3.1. Why Validation Matters: Regulatory, Scientific, and Clinical Perspectives

From a regulatory point of view, key bodies such as the FDA and EMA mandate robust validation. This is necessary to ensure patient safety and device efficacy before market approval. AI algorithms are classified as medical devices. Hence, validation must demonstrate both analytical and clinical validity [13,34,38,41]. This requires adherence to standards like ISO 13485 [42] and frameworks for Good Machine Learning Practice (GMLP). The adherence in turn ensures traceability, bias mitigation, and performance stability across diverse populations [11,13,41]. Without regulatory clearance, widespread clinical adoption remains impossible.

Scientifically, it is critical that AI claims are evidence-based. With this, validation moves beyond internal technical accuracy to confirming generalizability [6,9,10,11]. Generalizability is gauged through the algorithm’s ability to efficiently leverage independent, unseen datasets from different clinics, patient demographics, and laboratory protocols. In other words, generalizability evaluates AI’s predictive accuracy: the ability to identify true biological signals or if it merely overfits to artifacts in the training data. To ensure better outcomes, relevant validation studies ought to be prospectively designed to avoid inflated results [7,10,11]. Reproducibility is paramount for scientific acceptance [9,43].

Clinically, key stakeholders require proof of clinical utility. This refers to AI’s ability to actually improve patient outcomes or operational efficiency when compared to current practice [13,35,44,45,46]. Relevant validation must show that a given AI tool integrates seamlessly into workflows. That is, it does not cause delays or errors and its predictions lead to better clinical decisions. Further, validation should also provide pertinent explanations to foster clinician confidence and appropriate human–AI collaboration [45,46,47]. On top of these, ethical validation is also crucial [48]. This ensures that tools do not perpetuate biases or exacerbate health disparities [38,47].

### 3.2. Review of Key Clinical Trials and Validation Studies

Available validation evidence ranges from retrospective analyses to prospective trials and real-world implementation studies. One large-scale retrospective validation study is by Rodriguez et al. [6]. Their study presents a massive external validation that entailed applying AI models (iDAScore v1, iDAScore v2 and KIDScore D5 v3) to 70,456 embryos from multiple external clinics. Pertinent findings of the study show high predictive power for blastocyst formation and implantation potential. Hence, the model significantly outperforms traditional morphology alone and shows generalizability beyond the development dataset. In another related large-scale study, Barnes et al. [15] validated non-invasive ploidy prediction on large external cohorts.

Besides the large-scale retrospective studies, there are also prospective observational studies. For instance, Sarandi et al. [7] showed iDAScore significantly correlated with implantation potential in routine practice. This provides preliminary real-world evidence. In another study, Gilboa et al. [45] evaluated the implementation of the AiVF Score. Their findings revealed that the algorithm improved data-driven decision-making and correlated with positive clinical outcomes.

In addition to those two, there are comparative studies, comparing AI versus embryologists. For instance, in their systematic review, Salih et al. [24] found that AI often matched or exceeded embryologist accuracy in predicting implantation, more so with time-lapse data. In another comparative study, Fitz et al. [8] showed that embryologists improved their selection of high-implantation potential embryos when aided by AI. Additionally, Kim et al. [31] prospectively evaluated trust and efficacy. They found that AI-assisted ranking is clinically effective.

Albeit limited, there are also randomized controlled trials, the gold standard of scientific evidence. One significant RCT is the SelecTIMO trial by Kieslinger et al. [39]. The large-scale RCT compares uninterrupted culture with TLM+AI selection vs. standard culture. The findings showed non-inferiority for the TLM+AI arm. However, they did not demonstrate superiority in LBR over standard culture with morphology [39]. This highlights the complexity of proving outcome improvement. With limited RCTs, more are needed, specifically focusing on AI-driven selection vs. expert embryologist selection for SET [10,34]. In their review, Tian et al. [10] emphasize the need for rigorous external validation of prediction models through meta-analysis, which can only be achieved by more RCTs.

There are also studies validating AI beyond embryo selection. For instance, AlSaad et al. [35] reviewed AI models predicting ovarian stimulation outcomes. Their findings revealed promising accuracy but variable generalizability. In another overview, Sergeev and Diakova [49] proposed advanced KPI frameworks specifically for validating IVF pregnancy prediction models. This stresses the need for standardized performance metrics.

### 3.3. Heterogeneity in Outcomes and Challenges in Protocol Standardization

The heterogeneity in outcomes is fostered by the lack of defined end points. Presently, studies are using varied primary endpoints. This includes attributes such as fertilization, blastulation, implantation, clinical pregnancy, ongoing pregnancy, and live birth. Live birth is the gold standard. However, it requires long follow-up and large cohorts [6,10]. Implantation rate is commonly used but it does not equate to live birth. Performance metrics also vary in studies. The common metrics include accuracy, sensitivity, specificity, AUC-ROC, and positive/negative predictive value (PPV/NPV). AUC is more common. However, it does not reflect clinical utility at specific decision thresholds [7,9,11]. PPV/NPV are clinically more relevant. The key limitation is that the values depend heavily on prevalence.

There is also the aspect of variable performance. As per existing evidence, AI models often show reduced performance when validated externally compared to internal results [9,10]. Performance can vary significantly between clinics. This is due to differences in patient populations, lab conditions, and imaging equipment [6,9,11,46].

Just like heterogeneity in outcomes, protocol standardization is affected by several factors. One of them is Data Acquisition and Annotation. The lack of standardization in image/video acquisition is evident in key factors like microscope settings, magnification, focal planes, and TLM intervals. This creates variability that impacts AI input data [11,13]. Embryo annotation suffers from subjectivity and inter-center variability. This affects training data quality and validation benchmarks [10,11,13]. Another influencer of protocol standardization is algorithm training and bias. Models trained on data from specific populations or clinics tend to not generalize well to others. This potentially introduces bias against underrepresented groups [11,38,47,50]. The third key influencer is clinical and laboratory protocols. Heterogeneity is evident in ovarian stimulation protocols, culture media, incubators, embryo transfer techniques, and freeze-all strategies. It significantly influences outcomes, making it difficult to isolate the impact of the AI tool itself [10,12,13]. The fourth factor is integration and workflow. Currently, there is a lack of seamless integration with existing Electronic Medical Records (EMRs) and Laboratory Information Management Systems (LIMS). This hinders smooth adoption and data flow for validation [13,44]. Another key challenge is the training of staff on interpreting and using AI outputs consistently [13,44].

### 3.4. Summary

Clinical validation is fundamental in revealing AI’s theoretical promise and its safe, effective, and equitable use in IVF. Significant progress has been made with large retrospective validations and prospective observational studies. However, challenges persist. This is more so regarding heterogeneity in outcomes, proving live birth rate improvement in RCTs, ensuring generalizability across diverse settings, and standardizing protocols. To address these challenges, collaborative efforts are necessary, particularly in developing universal standards for data acquisition and reporting [13,44] and between AI developers, embryologists, clinicians, ethicists, and regulators [12,38,41]. Coordination is also relevant to conduct robust multi-center RCTs with live birth endpoints [10,34] and prioritizing explainable AI [45,47]. It is only through rigorous and ongoing validation that AI can truly fulfill its potential to enhance IVF outcomes.

For efficient advancement, this field can borrow lessons from AI in non-human reproductive technologies. The development and validation of AI for embryo selection and oocyte assessment are also advancing rapidly in domestic animal-breeding programs. In these fields, large-scale, standardized data collection is more feasible. That enables robust model training and clearer validation of economic and reproductive outcomes. Further, the ability to perform experimental follow-ups that are not possible in human studies provides stronger causal evidence for AI predictions. Lessons from these models highlight the critical importance of standardized imaging protocols, large heterogeneous datasets, and outcome-linked validation—principles that the human IVF field must prioritize. This should be through consortium-based data sharing and rigorous, prospective trial design to overcome current generalizability challenges.

## 4. Limitations, Biases, and Ethical Considerations of AI in IVF: Navigating the Complex Landscape

The integration of AI into IVF is promising. However, it is marred by significant limitations, inherent biases, and profound ethical dilemmas. As such, a critical examination is essential to ensure responsible development and deployment.

### 4.1. Algorithmic Bias and Generalizability Concerns

One of the significant challenges undermining the reliability and fairness of AI in IVF is algorithmic bias. This bias often compromises generalizability. One core type of bias underlying the compromised outcomes is data-driven bias, also referred to as demographic bias. Fundamentally, AI models are trained on historical datasets. If these datasets underrepresent certain demographics, the resulting algorithms are likely to perform poorly or produce biased predictions for those groups [38,47,50].

The risk of algorithmic bias is not merely theoretical but has been empirically demonstrated in other high-stakes medical AI systems. A seminal study by Obermeyer et al. [51] audited a widely used commercial algorithm designed to identify patients for extra care. The algorithm was trained to predict future healthcare costs as a proxy for health needs. The audit revealed that at the same algorithm risk score, Black patients had 26.3% more chronic illnesses than White patients (4.8 vs. 3.8 distinct conditions). This significant disparity arose due to systemic inequalities in access. Further, the algorithm systematically underestimated the health needs of Black patients. The authors calculated that correcting this bias would increase the percentage of Black patients identified for extra care from 17.7% to 46.5%. This is a stark illustration of how biased training objectives can perpetuate and amplify healthcare disparities. While comprehensive public audits of IVF-specific AI platforms are still limited, this case highlights the critical importance of scrutinizing the objectives and training data composition of embryo selection algorithms. If such models are trained on datasets that over-represent certain demographics or use proxies for embryo viability that do not generalize equitably, they risk introducing similar biases. This will disadvantage underrepresented patient groups in their pursuit of fertility treatment.

The negative outcomes of this include the exacerbation of existing health disparities and disadvantaging underrepresented populations in access to optimal AI-assisted care [38,50]. The lack of diverse applicability is more evident in models trained primarily on data from high-resource settings or specific genetic backgrounds [11,38]. The second key type of bias is annotation bias. The ground truth, that is, the data used to train embryo selection models is often based on inputs by embryologists, which tend to be subjective. This inherent subjectivity and inter-observer variability [11] means that the training data itself is hallmarked by human bias, which the AI can amplify [11,51]. Further, outcomes are influenced by factors beyond embryo quality such as endometrial receptivity and transfer technique. This creates noisy labels that complicate learning true embryo potential [52,53,54].

The third type of bias is technical and environmental bias. As established earlier, there exist key variations in laboratory protocols, imaging equipment, and image acquisition techniques. These fundamental variances introduce technical heterogeneity [11]. AI models trained on data from one specific technical environment may fail to generalize to others [55]. As such, this limits their broad applicability and requires costly local retraining [11,38,53].

The fourth key issue is opaqueness, otherwise labeled as the black box problem and the lack of explainability. Many complex AI models, particularly deep learning systems, function as black boxes. This makes it difficult to understand why a specific prediction was made [37,38,47,56,57,58]. This lack of explainability hinders trust, making it challenging to identify and correct biases embedded within pertinent models. It also complicates regulatory oversight [37,38,56,58]. The overall effect of this dilemma is that clinicians and patients tend to struggle linking AI decisions with human judgment or biological understanding [46,57].

Overall, it is evident that the lack of explainability fundamentally hinders clinical trust and the ability to identify embedded biases. However, recent advances in Explainable AI (XAI) offer promising pathways to demystify these complex models. Techniques such as SHapley Additive exPlanations (SHAP) and Local Interpretable Model-agnostic Explanations (LIME) can be applied to embryo image analysis. These methods generate visual attribution maps that highlight which specific regions of a static or time-lapse embryo image such as the texture of the cytoplasm or the thickness of the zona pellucida, that most strongly influenced the model’s prediction of viability, ploidy, or implantation potential. By visualizing these ‘attention’ regions, XAI can transform an opaque prediction into an interpretable assessment, allowing embryologists to reconcile AI outputs with established morphological knowledge, identify potential model errors, and build a collaborative, evidence-based decision-making process.

The fifth and last key issue linked with generalizability discrepancies is overfitting and limited external validation. Most models have been shown to have poor external validation [9,11]. This lack of robustness and generalizability is a major limitation for clinical adoption [11,38,53]. Continuous validation across diverse settings is crucial. Nevertheless, it is resource-intensive [38,53].

### 4.2. Ethical Dilemmas in Automated Embryo Selection

The automation of embryo ranking and selection using AI raises profound ethical questions [48]. One key area influenced is autonomy, agency, and shared decision-making. AI recommendations are likely to overshadow patient values and preferences in embryo selection [59,60,61,62]. Fundamentally, patients may feel pressured to accept AI rankings without fully understanding pertinent limitations or certain algorithmic values. This potentially undermines their autonomy and the shared decision-making process critical to reproductive care [59,60,61,63]. It is important that stakeholders ensure patients retain meaningful choice and understand key limitations of AI predictions [56,59,60].

The second key ethical concern is responsibility and accountability. AI systems, despite their efficiency, are likely to recommend an embryo that fails to implant or results in miscarriage. Conversely, an embryologist is likely to override an AI recommendation that might have succeeded. Who assumes accountability in these scenarios? [38,47,52,57]. The complex interplay between algorithm developers, clinicians, embryologists, and the technology itself creates ambiguity in assigning responsibility for outcomes. Hence, it raises significant medico-legal concerns [38,47,57,64].

The third key dilemma is the dehumanization and the commodification of embryos, which strengthens the eugenics concern. Over-reliance on algorithmic scoring risks reducing human embryos to mere numerical values or data points. This potentially fosters a dehumanized view of early human life and the IVF process [52,62,65]. Principally, the commodification clashes with the deeply personal, emotional, and often ethically charged nature of creating and selecting embryos for potential life [61,62,65]. There is a symbolic meaning attributed to embryos by patients given the sacred nature of life. This may be disregarded by purely algorithmic assessments [62,65].

The fourth concern revolves around defining what is optimal and providing value judgements. AI models are trained to objectively predict outcomes like implantation or live birth. These outcomes reflect specific values, the most common being maximizing the success rate per transfer. However, the values may not align with patient priorities, which tend to vary and include examples such as minimizing miscarriage risk or ethically objecting to discarding embryos as determined by the algorithm [59,60,61,62]. The discrepancy emanates from the reality that the algorithm inherently embeds the values of its designers and the data it was trained on [38,47,60].

The last critical concern is the impact on professional judgment and expertise. There is a possibility that widespread AI adoption could potentially deskill embryologists. This is most likely to be through reducing opportunities for developing and applying nuanced morphological assessment expertise [52,57,66]. Further, Toschi et al. [46] highlight the tension between AI recommendations and embryologist/clinician judgment. This is likely to create ethical and practical dilemmas regarding when to override the algorithm and who bears the burden of that decision [46,52,57,66].

### 4.3. Data Governance, Privacy, and Informed Consent Issues

Vast amounts of sensitive data are required to develop and operate AI in IVF. As such, this necessitates robust governance frameworks. One key area is sensitivity of reproductive data. IVF involves exceptionally sensitive personal data. This includes detailed medical histories, genetic information, and highly personal outcomes such as pregnancy success or miscarriage [63,64,67,68,69]. If this information falls in the wrong hands through unauthorized access and breaches, its misuse carries severe consequences for patient privacy, dignity, and psychological well-being [38,64,67,68]. Regarding informed consent, obtaining true consent for using patient data in AI development and deployment is complex [59,63,67,69]. It entails several significant attributes that patients must first understand. Firstly, patients need to know how their data will be leveraged in training, validating, or operating AI systems. Secondly, individuals need to grasp the potential risks such as data breaches and secondary uses of data that include research and commercial development. Thirdly, it is prudent for patients to understand the limitations and potential biases of the AI tools used in their care. Fourthly, patients must know their rights regarding data access, correction, and withdrawal [59,63,64,67,69]. Currently, the consent processes upheld often lack the specificity and transparency required for AI applications [63,64,67].

Regarding data ownership and control, ambiguity persists regarding who owns the sensitive data generated during IVF cycles. The key stakeholders in contention include patients, clinics, and the technology providers collecting and processing the information [63,64,66]. Patients often have limited control over how their data is shared. This is more particular when it comes to commercial AI development [38,64,66,68]. It is thus relevant to have clear governance structures defining data ownership, stewardship, and patient rights [64,66,69].

On data security and breach risks, the centralized storage of large, sensitive datasets makes them significant targets for malicious intents [64,68,69]. Hence, robust cybersecurity measures are non-negotiable. However, breaches remain a significant risk with potentially devastating consequences for patients [38,64,68]. Regarding the uses of collected data, it is likely to be leveraged for other purposes like furthering AI research or product development by private companies. This is often without explicit patient consent or benefit-sharing [64,66,68]. Stakeholders need to ensure safeguards against the unauthorized commercial exploitation of sensitive reproductive data [38,63,64,66]. Lastly, there is the key issue of lacking standardized data formats and interoperable systems that tend to create data silos. This hinders the development of efficient AI models while simultaneously complicating patient data portability and access [53,66,69].

### 4.4. Summary

Overall, the integration of AI into IVF offers transformative potential. However, it is inextricably linked to significant limitations, biases, and ethical complexities. Algorithmic bias threatens equity and generalizability. On the other hand, the lack of transparency undermines trust and accountability. There are also ethical dilemmas concerning autonomy, responsibility, dehumanization, and the definition of optimal. Hence, robust data governance, ensuring true informed consent, strict privacy provisions, clear data ownership, and heightened security are fundamental for the field’s overall efficiency.

A multifaceted approach is crucial to addressing the key dilemmas and operational limitations. For starters, stakeholders need to foster transparency and bias detection/mitigation techniques [37,56,58]. Further, clinicians, patients, and regulators need to develop clear ethical guidelines and accountability frameworks [38,47,59,61]. Additionally, stringent and transparent data governance protocols prioritizing patient rights need to be adopted [64,67,69]. Ignoring these limitations and ethical dimensions has severe repercussions. It risks undermining patient trust, exacerbating inequalities, and causing harm. These significantly limit the very innovation that promises to advance reproductive medicine [38,52,64,66].

## 5. Pathways to Clinical Integration of AI in IVF: From Adoption to Optimization

The successful integration of AI into routine IVF practice depends on several aspects beyond just technological capability. Pertinent stakeholders need to foster strategic implementation, workforce development, and seamless technical compatibility. Covered in this segment of the review are the critical pathways to efficient clinical integration.

### 5.1. Practical Strategies for Integrating AI into IVF Clinics

One significant strategy is through phased, goal-oriented implementation. This entails starting small and clearly defining the key objectives. For instance, clinics can begin with a pilot program with specific targets such as AI-assisted embryo selection [36], which is more efficient than attempting full-scale integration [33,40,70]. For success, it is relevant to define clear and measurable goals. Key examples include improving consistency, reducing time-to-pregnancy, increasing SET rates, and optimizing resource use [34,53,71]. Beyond goal establishment, conducting a needs assessment and selecting a vendor is crucial. During the needs assessment, clinics need to evaluate AI models based on the following key factors. These include proven clinical validation, compatibility with existing equipment, interoperability potential, cost-effectiveness, vendor support, and transparency features [33,53,72,73,74]. Solutions with a proven track record need to be prioritized [27,33].

After selecting an AI solution, the next key aspect is workflow integration and changing management. Clinics need to map existing workflows meticulously and design the AI tool without key operational dilemmas [33,72,73,75]. Regarding changing management, this needs to be actively accomplished. For instance, key stakeholders such as embryologists, clinicians, and nurses need to be involved from the beginning. Key concerns need to be addressed transparently and the AI’s value proposition clearly demonstrated [33,40,66]. Pilot testing as established earlier is crucial to refine integration [33,76].

In addition to the above is fostering clear governance and protocols. These are necessary to define the AI’s key roles. For instance, will the AI’s input be mandatory or advisory and how will AI/embryologist judgment conflicts be resolved? [33,40,76]. Further, clinics need mechanisms to continuously monitor AI performance and clinical impact [33,53,71,76]. The last aspect is the pilot-to-scale approach. It is only after a successful pilot that has informed pertinent adjustments that the AI solution can be scaled across the clinic [33,74,76].

### 5.2. Training and Upskilling of Healthcare Professionals

As per the current evidence, a significant percentage of embryologists lack key AI literacy skills such as interpreting confidence scores [77,78]. Further, a significant portion of clinicians struggle to reconcile AI outputs with clinical context [40]. Hence, their training is critical and it must move beyond technical proficiency to critical evaluation. This means empowering staff to conceptualize key fundamentals. These include basic AI/ML concepts and the purposes, strengths, and limitations/biases of a given algorithm [40,53,66,77,78,79]. This ensures transparency as much as possible. They need to know how to critically interpret AI results within the clinical context. Additionally, they also need to understand probabilities and confidence intervals rather than absolute truths [40,66,77,79]. Training should emphasize AI as a decision support tool and not a replacement for clinical expertise and judgment [33,40].

Another relevant consideration is that they need to know how to identify errors and limitations. These include potential technical failures, data input errors, and outputs that seem biologically implausible or contradict strong clinical indicators [53,66,79]. Moreover, they need to know how to efficiently leverage AI in clinical reasoning, combining its insights with patient history, clinical findings, morphology assessment, and patient values [40].

Besides enhancing critical evaluation, their training also needs to be tailored and multidisciplinary. For embryologists, the focus should be on AI-assisted embryo assessment interpretation, quality control flags, understanding algorithm training data limitations, and workflow integration within the lab [53,66,73,79]. For clinicians, the spotlight should be on interpreting predictive outputs such as ovarian response. This will ensure that they are efficiently integrated into treatment planning and patient counseling. They will also understand the pertinent ethical implications [66,77,78,79]. For all staff, the training should touch on data privacy, security protocols related to AI systems, and basic troubleshooting [76,79,80].

Lastly, the training needs to be continuous and competency assessed. AI evolves rapidly. Continuous training is necessary to update staff on software upgrades, new validation data, and emerging best practices [77,78,79]. On top of training, competency in using and interpreting AI tools needs to be regularly assessed [78,79]. Further, clinics need to establish a culture of feedback where staff report challenges and insights from using AI in practice [33,40,66].

### 5.3. Interoperability with Existing Infrastructure and EMRs

Seamless data flow is crucial for efficient AI integration. It is also necessary to avoid redundant data entry, a major barrier to adoption [33,53,72,74,76,80]. AI tools need access to diverse data. The sources of this data include patient demographics, stimulation protocols, lab results, ultrasound images, TLM videos, and embryology annotations and outcomes. Those various data sources are available in several systems that include EMRs, Laboratory Information Management Systems (LIMS), TLM incubators, imaging archives, and PGT labs [72,73,75,76,80]. Lack of interoperability creates data silos. These hinder potential AI performance, increase workload, and risk errors [33,53,80].

To enhance connectivity, clinics must first adopt standards like HL7 FHIR (Fast Healthcare Interoperability Resources), necessary for structured data exchange and DICOM for medical imaging [72,76,80]. This facilitates communication between different systems. They also need to utilize application programming interfaces (APIS). These allow for secure, programmable data exchange between the AI platform and the clinic’s EMR, LIMS, and TLM systems. In turn, the exchange enables automated data retrieval and result return [74,76,80]. They also need to implement integration engines or middleware platforms that act as central hubs. These translate data between different systems that use different formats or protocols [76,80]. Ensuring the successful implementation can simplify connectivity, especially in complex legacy environments. Clinics can also consider cloud-based AI platforms that offer easier integration via APIs. They also reduce on-premise IT burden. Additionally, on top of security and regulatory compliance, hybrid models can be leveraged to offer a balance [74,76,80]. Lastly, IoT can be leveraged to improve data flow [80].

### 5.4. Summary

The pathway to successful AI integration in IVF clinics is multifaceted. It necessitates a strategic and phased implementation approach, which ought to focus on solving specific clinical problems alongside robust governance. A crucial determining factor is investing in comprehensive, ongoing training. This is necessary to empower healthcare professionals to be efficient users of AI. Finally, given interoperability challenge, adopting standards, APIs, and middleware is fundamental to unlocking AI’s full potential. It will also ensure data accuracy and streamline workflows. To efficiently overcome interoperability hurdles, clinics, AI developers, EMR/LIMS vendors, and standards bodies must collaborate [33,74,80]. Generally, by ensuring practical implementation, workforce development, and technical integration, IVF clinics can harness AI, leveraging it to enhance precision, efficiency, and patient outcomes while upholding key ethical provisions [27,40,71].

## 6. Case Studies: Real-World Applications and Outcomes of AI in IVF

Despite ongoing key developments, AI has been adopted in IVF clinics, yielding valuable insights through real-world implementations. This segment of the review, primarily through Table 2 below, provides an evidence-based analysis of outcomes, regional adoption patterns, and critical barriers/facilitators.

### 6.1. Clinic-Level Success Stories and Comparative Outcomes

Overall, concerning accuracy gains, there has been a consistent improvement in embryo viability prediction compared to traditional methods by a margin of 10–25% [8,23,24,31,33,36]. In efficiency, there has been a 30–50% reduction in embryologist time per embryo cohort [23,33,36,40,53]. In terms of impact on outcomes, early adopters report 5–15% increase in cumulative live birth rates (CLBR) [12,32].

### 6.2. Lessons from Early Adopters: Regional Perspectives

The United States (US) is one of the early adopters. Key motivators or AI adoption are mainly market competition and patient demand for innovation [32,40]. Core operational challenges as acknowledged in the literature include reimbursement dilemmas given that this is an emerging area. As such, patients often bear pertinent AI costs, which are expensive [32,82]. Despite the challenges, success stories have been recorded. For instance, the review by Riegler et al. [33] shows that some centers significantly reduced multiple pregnancies using AI-guided SET. Alongside the US, the European Union (EU) is also one of the early adopters. Here, the spotlight has been more on the regulatory outlook. For instance, proof of added clinical value must be confirmed before adoption [83,84]. Implementation-wise, phased integration with audit trails such as the UK’s HFEA requirements are necessary [44,84]. Regardless, success stories have also been recorded. For instance, in an overview involving embryologists from Korea, Malysia, and the US, AI reduced embryo disposal errors by a margin of 15% [31]. In Asia, the uptake has been significant in Japan, China, and South Korea, an occurrence that is significantly attributed to government initiatives such as Korea’s Bio-Health 2030 [31,74]. The primary focus as evidenced in these countries has been on image-heavy platforms compatible with high-volume clinics [31,74]. Success has also been recorded, as has been the case with other early adopters. For instance, in their literature, Yao et al. [81] note that AI doubled triage efficiency in US IVF centers.

The critical takeaways from the above three early adopters is that staged implementation is efficient, transparency in outcomes helps build trust, and aligning with relevant regulations as seen in the case of the EU is crucial.

### 6.3. Barriers and Facilitators to Adoption

A key barrier is cost, with upfront cost running in excess of USD 200 000 per AI system [32,82]. Even if it is not full-on adoption, 68% of clinics cite high subscription fees in excess of USD 20k a year as an adoption hurdle [32,82]. Given the unclear return on investment, most clinics remain hesitant in adopting relevant AI systems [18,81]. Another barrier is resistance by embryologists due to job security concerns, as evidenced by 42% of clinics in the EU [82,83]. Further, the lack of transparency makes a large percentage of clinicians distrust AI recommendations [8,31]. This also affects patients, as many of them express unease about AI decision-making without human intervention [31,85]. Technically, system incompatibility is a major issue [12,81].

On facilitators, transparency remains a key factor. Clinics adopting platforms with published real-world outcomes such IVY’s open performance dashboards exhibit a high rate of acceptance [31,40]. Further, hybrid decision-making, balancing AI’s input with embryologists’ experience, is another facilitator. This reduces resistance [8,12,40]. In addition to these, there is also regulatory alignment. For instance, in the EU, clinics using ESHRE-compliant AI tools report smoother adoption [83,84]. Cost-wise, given that it is a major barrier, facilitation is evident through the phased cost models where clinics pay per cycle. This has increased adoption in medium-sized clinics [12,32].

Overall, in the overview of real-world applications, key takeaways include the relevance and necessity of human–AI synergy. Success stories consistently feature AI as a decision support tool with embryologists validating outputs [8,24,40]. Secondly, contextualization matters. Center-specific customization as noted in Yao et al. [81] outperforms generic models. Thirdly, there is a significant gap in patient counseling, with only a small percentage of clinics having protocols in place to explain AI use to patients [85]. Fourthly, top-performing clinics continuously retrain AI with local data. A good example is the Boston IVF model that updates quarterly [81].

## 7. Future Directions and Policy Recommendations for AI in IVF: Toward Precision, Equity, and Governance

### 7.1. Innovations on the Horizon

#### 7.1.1. AI-Enhanced Genomics and Multi-Omics Integration

One of the key developments needed is Polygenic Risk Scoring (PRS—defined in Appendix A). This is an AI algorithm that integrates genomic data with embryo morphology. In doing so, it predicts inherited disease risks and developmental potential, thus reducing reliance on invasive PGT-A [12,27,50]. The second key area is non-invasive embryo analysis. This combines real-time metabolomics and proteomic sensors in culture media with AI for viability assessment. As a result, it enables continuous embryo health monitoring [3,86,87]. The third key enhancement entails endometrial receptivity clocks. With these, deep learning models analyze endometrial RNA-sequencing data. This is necessary to pinpoint optimal implantation windows [3,27,50].

#### 7.1.2. Real-Time Adaptive Systems

A key advancement under these systems would be closed-loop ovarian stimulation. This combines IoT-enabled hormone sensors with AI adjusting medication doses in real time. This helps prevent OHSS and maximize oocyte yield [27,76,80]. The second key area is automated quality control. This entails leveraging computer vision to monitor lab conditions such as temperature and pH with instant alerts whenever an anomaly is detected [3,27,53].

#### 7.1.3. Predictive Patient Journey Mapping

Lifetime fertility forecasting is the key advancement necessary to better outcomes. AI synthesizing genetic, lifestyle, and clinical data can be leveraged to effectively predict future fertility decline and guide proactive interventions [27,50,88].

### 7.2. Recommendations for Stakeholders

Researchers are the main key stakeholders. Hence, they must prioritize fostering explainable AI (XAI—defined in Appendix A). To achieve this, they ought to develop interpretable models such as SHAP and LIME to demystify embryo selection logic [37,56,58]. Researchers also need to address bias proactively. This can be achieved by using federated learning across diverse populations, which is necessary to minimize ethnic and socioeconomic disparities [38,47,50]. Thirdly, they need to focus on live birth endpoints. This mandates shifting validation from embryo rankings to cumulative live birth rates (CLBR) in RCTs [10,39]. On top of these is the aspect of data heterogeneity, that is, variations in microscope models, imaging settings, focal planes, and lab protocols that severely limit model generalizability. Future development ought to prioritize robust technical strategies for domain adaptation. Rather than requiring standardized imaging across all clinics, AI models can be engineered to be invariant to these technical confounders. Some of the promising approaches to achieve this include test-time adaptation and feature alignment. Models could be designed to dynamically adjust to the input characteristics of a new clinic using unsupervised alignment techniques. This has the potential to reduce performance drop-off during external validation. Another approach is the domain-invariant representation learning. Techniques such as those employing generative adversarial networks can be used to learn embryo features that are discriminative for viability but invariant to the imaging ‘domain’. Adversarial training can encourage the model to extract features that cannot be used to predict the source clinic. The outcome is an improved generalizability to new, unseen laboratory environments.

Besides researchers, policymakers are the second most crucial stakeholders. To enhance AI in routine IVF, they need to ensure unified regulatory frameworks. A better way is to establish FDA/EMA joint guidelines for SaMD validation, requiring real-world performance monitoring [13,41], bias audits in training data [41,47], and transparency in algorithm updates [41,89]. They also need to work on reimbursement models by creating CPT codes for AI-assisted procedures to improve access [32,71]. A feasible example would be Medicare coverage for AI-guided SET. Thirdly, policymakers need to establish data sovereignty laws. These provisions will help mandate patient ownership of reproductive data with opt-in consent for research [59,64,69].

The third key stakeholders are clinicians. As major players in the adoption of AI in IVF, they need to establish hybrid decision protocols by implementing frameworks where AI suggestions require embryologist validation [8,33,40]. Secondly, given the fast developments taking place, they need continuous AI literacy training to efficiently integrate AI interpretation into REI fellowship curricula [77,78,79]. Thirdly, they need to foster patient-centric transparency. This can be best achieved by disclosing AI limitations in consent forms [63,67,69].

### 7.3. Global Standards and Interdisciplinary Collaboration

Key urgent needs include data interoperability. Stakeholders need to adopt universal HL7 FHIR standards for IVF data exchange, thus efficiently enabling cross-border AI training [72,74,80]. Validation benchmarks are also needed, specifically through an international consensus on KPIs [9,11,34]. Thirdly, ethical guardrails are critical. A sufficient example is a UNESCO-led global charter prohibiting AI for designer embryo selection [52,61,65].

### 7.4. Collaborative Frameworks

Several key initiatives are required. The first one is a global IVF AI consortium. This is required to pool de-identified data from over 500 clinics. The consortium is also necessary to develop open-source reference models. The second key initiative is fertility AI ethics review boards. These are relevant to audit algorithms for bias, as well as oversee and guide patient data usage. The third relevant initiative to advance AI in IVF entails cross-industry partnerships. For instance, tech giants such as NVIDIA can collaborate with biotech firms to co-develop secure cloud platforms.

### 7.5. The Path Forward

In the short run, that is, within 3 years, stakeholders need to validate AI-genomics integration through key trials such as Polygenic IVF Efficacy Trial (PIVET) [12,50]. They also need to launch a clinician certification in AI interpretation program [78,79]. In five years’ time, mandatory AI registries need to be implemented to track real-world outcomes [13,41]. In the long run, that is, over 5 years, stakeholders need to realize precision IVF. This is where fully personalized protocols from stimulation to transfer via adaptive AI are leveraged for efficiency [3,27,50].

## 8. Conclusions

Artificial Intelligence promises to transform IVF from an artisanal practice into a more precise discipline. This mainly by offering superior capabilities in embryo selection, workflow standardization, and outcome prediction. Existing evidence asserts that AI has the potential to achieve the following: enhance the accuracy of identifying viable embryos by 10–25%, significantly reduce embryologist workload (by 30–50%), and advance elective single-embryo transfer (eSET–defined in Appendix A) through superior, consistent viability prediction. Innovations in AI-enhanced genomics and real-time biosensors could further personalize care. However, the definitive impact on per-transfer live birth rates requires further validation through robust RCTs. This is because the current evidence points more strongly towards gains in process efficiency, consistency, and the reduction in multiple pregnancies.

Despite the promise, AI in IVF necessitates careful adoption. One critical consideration is evidence-based adoption. Retrospective studies such as Rodriguez et al.’s [6] 70,456-embryo analysis demonstrate technical feasibility. However, prospective RCTs proving live birth improvements are urgently needed. It is imperative that AI tools evolve from just predictive curiosities to clinically indispensable assets. This can be achieved through standardized KPIs for cross-platform benchmarking, real-world performance registries and continuous recalibration with local data. The second critical consideration besides evidence-based adoption is ethical vigilance. Key attributes to be fostered include bias mitigation through federated learning across diverse populations to prevent algorithmic discrimination, transparency through XAI frameworks demystifying embryo scoring, and patient sovereignty through clear consent protocols for data usage and AI-assisted selection. The third critical consideration is global collaboration. This needs to be achieved through unified data standards such as HL7 FHIR for IVF to enable interoperable systems, international regulatory harmonization such as FDA and EMA joint SaMD guidelines, and knowledge-sharing platforms such as a WHO-led IVF AI consortium.

The path forward demands augmented intelligence where AI elevates human expertise through hybrid decision-making, continuous upskilling, and patient-centered focus. Simply put, “AI will not replace embryologists, but embryologists using AI will replace those who don’t.”

As it stands, it is imperative that stakeholders harness AI’s power to alleviate infertility suffering. This needs to be accomplished while vigilantly safeguarding human dignity, equity, and the sacred trust inherent in reproductive care. It is only through principled innovation anchored in evidence, ethics, and global solidarity that we can fully ensure these technologies fulfill their transformative potential.

## Figures and Tables

**Table 2:** Case studies of real-world implementations of AI in IVF.

Study/Clinic	AI Platform	Key Findings
[8]—Multi-center	Proprietary Algorithm	Embryologists’ accuracy in selecting high-implantation embryos increased by approximately 12% when using AI assistance. Human–AI collaboration outperformed each intervention when applied alone.
[31]—Seoul	ResNet50	The accuracy of the embryologists was 34 (38%) and that of the AI model was 59 (66%). When the embryologist was guided by the AI score, the accuracy rate increased to 45 (50%). The AI model outperformed embryologists in selecting an embryo that led to pregnancy by 25 (28%), and embryologists with AI guidance outperformed embryologists without AI guidance by 11 (12%).
[24]—Systematic Review	Several platforms	The analysis indicates that when comparing the accuracies of AI models against embryologists, all the 20 studies reported a better performance in favor of the AI models by at least 4% higher prediction accuracy. In one of the studies, AI outperformed the embryologists by 45% when correlating embryo quality with implantation outcome.
[81]—US	Center-Specific ML	Machine learning, center-specific (MLCS) models significantly improved minimization of false positives and negatives overall (precision recall area-under-the-curve) and at the 50% live birth prediction threshold.
[7]	iDAScore	The iDAScore of blastocysts that implanted was significantly higher than those that did not implant.

## Data Availability

No new data were created or analyzed in this study. Data sharing is not applicable to this article.

## Abbreviations

The following abbreviations are used in this manuscript:

AFC	Antral Follicle Count
AI	Artificial Intelligence
AUC	Area Under the Curve (a statistical measure of model performance)
AUC-ROC	Area Under the Receiver Operating Characteristic Curve
BMI	Body Mass Index
CLBR	Cumulative Live Birth Rate
CNN	Convolutional Neural Network (a type of AI for image analysis)
CPT	Current Procedural Terminology (US medical billing codes)
DL	Deep Learning
eSET	elective Single-Embryo Transfer
EMA	Embryo Multi-dimensional Analysis (AIVF’s platform name)
EMR	Electronic Medical Record
ERICA	Embryo Ranking Intelligent Classification Algorithm
ESHRE	European Society of Human Reproduction and Embryology
EU	European Union
FDA	Food and Drug Administration (US regulatory body)
FHIR	Fast Healthcare Interoperability Resources (a data standard)
GMLP	Good Machine Learning Practice
GDPR	General Data Protection Regulation (EU data privacy law)
HIPAA	Health Insurance Portability and Accountability Act (US data privacy law)
HL7	Health Level Seven (a healthcare data standards organization)
ICM	Inner Cell Mass
ICSI	Intracytoplasmic Sperm Injection
IoT	Internet of Things
ISO	International Organization for Standardization
IVF	In Vitro Fertilization
KPI	Key Performance Indicator
LBR	Live Birth Rate
LIME	Local Interpretable Model-agnostic Explanations (an XAI technique)
LIMS	Laboratory Information Management System
ML	Machine Learning
NIPA	Non-Invasive Ploidy Assessment
OHSS	Ovarian Hyperstimulation Syndrome
PGT-A	Preimplantation Genetic Testing for Aneuploidy
PPV	Positive Predictive Value
NPV	Negative Predictive Value
PRS	Polygenic Risk Scoring
RE	Reproductive Endocrinologist
REI	Reproductive Endocrinology and Infertility (a medical subspecialty)
RCT	Randomized Controlled Trial
RNN	Recurrent Neural Network (a type of AI for sequence data)
SaaS	Software as a Service
SaMD	Software as a Medical Device
SET	Single-Embryo Transfer
SHAP	SHapley Additive exPlanations (an XAI technique)
TE	Trophectoderm
TLM	Time-Lapse Microscopy
US	United States
WHO	World Health Organization
XAI	Explainable Artificial Intelligence

## Appendix A. Glossary of Terms

**A**
**Algorithm**: A set of rules or instructions followed by a computer to perform calculations or solve problems. In AI, algorithms learn patterns from data to make predictions or decisions.
**Aneuploidy:** The condition of having an abnormal number of chromosomes in a cell. Embryos with aneuploidy often fail to implant or result in miscarriage.
**Annotation (Data Annotation):** The process of labeling data (example—images of embryos) with tags to provide context and meaning, which is used to train AI models. In embryology, this involves an embryologist labeling an embryo’s stage, grade, or outcome.
**B**
**Blastocyst:** An advanced stage of embryo development, typically reached by day 5 or 6 after fertilization. It is characterized by a fluid-filled cavity and two distinct cell types: the inner cell mass (which becomes the fetus) and the trophectoderm (which becomes the placenta).
**Bias (Algorithmic Bias):** Systematic and repeatable errors in an AI system that create unfair outcomes, such as privileging one arbitrary group of users over others. Often results from unrepresentative training data.
**C**
**Computer Vision:** A field of AI that enables computers and systems to derive meaningful information from digital images, videos, and other visual inputs. It is core to AI embryo analysis.
**Clinical Validation:** The process of assessing whether an AI tool performs accurately, safely, and effectively in a real-world clinical setting, not just in a lab.
**Cumulative Live Birth Rate (CLBR):** The probability of achieving a live birth from one complete IVF cycle, which may include the transfer of all fresh and frozen embryos derived from a single ovarian stimulation. It is a key endpoint for measuring IVF success.
**D**
**Deep Learning (DL):** A subset of machine learning that uses multi-layered (deep) neural networks to analyze complex patterns in large amounts of data. It is particularly powerful for image recognition tasks like embryo analysis.
**Dehumanization (Ethical):** The ethical concern that reducing human embryos to numerical scores or data points by AI could undermine the personal, emotional, and sacred value attributed to them by patients.
**E**
**Elective Single-Embryo Transfer (eSET):** The practice of transferring one high-quality embryo to the uterus to achieve a singleton pregnancy, thereby avoiding the risks associated with multiple pregnancies (twins, triplets). AI aids in selecting the single best embryo for eSET.
**Embryologist:** A scientist and medical professional who specializes in the handling and assessment of eggs, sperm, and embryos in the IVF laboratory.
**Endometrial Receptivity:** The state of the uterine lining (endometrium) when it is ready for an embryo to implant. The “window of implantation” is a short period when receptivity is optimal.
**Explainable AI (XAI):** A set of processes and methods that allows human users to understand and trust the results and output created by machine learning algorithms. Crucial for building clinician trust in AI recommendations.
**F**
**Federated Learning:** A decentralized machine learning technique where an algorithm is trained across multiple decentralized devices or servers holding local data samples, without exchanging them. This helps improve privacy and reduce bias by leveraging diverse datasets.
**G**
**Generalizability:** The ability of an AI model to perform accurately on new, unseen data that comes from a different but related distribution to its training data (e.g., from a new clinic with different protocols).
**Ground Truth:** The data that is known to be accurate and is used to train and validate an AI model. In embryo selection, this is often the clinical outcome (e.g., implantation, live birth) associated with an embryo’s data.
**I**
**Inner Cell Mass (ICM):** The cluster of cells inside a blastocyst that will develop into the fetus. One of the key morphological features graded by embryologists and analyzed by AI.
**Interoperability:** The ability of different information systems, devices, and applications (e.g., EMR, LIMS, AI software) to access, exchange, and use data in a coordinated manner. It is a major technical hurdle for AI integration.
**L**
**Live Birth:** The birth of one or more living infants. It is considered the gold-standard endpoint for measuring the success of an IVF treatment.
Laboratory Information Management System (LIMS): A software-based solution that supports the operation of a modern laboratory by managing samples, associated data, and workflows.
**M**
**Machine Learning (ML):** A subset of AI that provides systems the ability to automatically learn and improve from experience without being explicitly programmed. It focuses on the development of algorithms that can analyze data and make predictions.
Morphokinetics: The timing of key developmental events in an embryo (e.g., time to division into 2 cells, 3 cells, etc.), captured by time-lapse microscopy.
**Morphology:** The visual assessment of an embryo’s form, structure, and cellular characteristics (e.g., cell size, symmetry, fragmentation) under a microscope.
**N**
**Non-Invasive Ploidy Assessment (NIPA):** The use of AI to predict the chromosomal status (ploidy) of an embryo by analyzing time-lapse images or other non-invasive markers, potentially reducing the need for invasive PGT-A biopsy.
**O**
**Ovarian Hyperstimulation Syndrome (OHSS):** A potentially serious complication of IVF treatment where the ovaries become swollen and painful due to an excessive response to hormone stimulation drugs. AI can help personalize dosing to minimize this risk.
**P**
**Preimplantation Genetic Testing for Aneuploidy (PGT-A):** A technique used during IVF to genetically test embryos for chromosomal abnormalities before transfer. It involves a biopsy of cells from the embryo.
**Polygenic Risk Score (PRS):** A number that summarizes the estimated effect of many genetic variants on an individual’s phenotype, typically related to their risk of developing a particular disease. An emerging AI application to predict inherited risks in embryos.
**S**
**Software as a Medical Device (SaMD):** Software intended to be used for one or more medical purposes without being part of a hardware medical device. AI algorithms for embryo selection are classified as SaMD and are regulated by bodies like the FDA and EMA.
**T**
**Time-Lapse Microscopy (TLM):** Technology that involves an incubator with a built-in microscope that takes frequent images of developing embryos without removing them from the optimal culture environment. This generates the video data used by AI algorithms.
Trophectoderm (TE): The outer layer of cells in a blastocyst that is responsible for implantation and develops into the placenta. Its quality is a critical factor in embryo selection.
**X**
**XAI:** See Explainable AI.
